# Sodium-Glucose Transport Protein 2 Inhibitors Association with Euglycemic Diabetic Ketoacidosis

**DOI:** 10.1155/2023/6835882

**Published:** 2023-02-27

**Authors:** Caroline Wojtas, Alex P. Rasarmos, Naja Naddaf

**Affiliations:** ^1^OMS-III, Edward via College of Osteopathic Medicine–Auburn, Auburn, AL 36830, USA; ^2^OMS-IV, Lake Erie College of Osteopathic Medicine, Bradenton, FL 34211, USA; ^3^Orange Park Hospital, Orange Park, FL 32073, USA

## Abstract

Diabetic ketoacidosis (DKA) is a life-threatening medical emergency that occurs in both type 1 and type 2 diabetes mellitus. Here, we describe the case of a 49 year-old male patient with type 2 diabetes mellitus who presented to the emergency department with complaints of epigastric abdominal pain and intractable vomiting. He had been on sodium-glucose transport protein 2 inhibitors (SGLT2i) for 7 months. Considering the clinical exam and lab findings with a glucose level of 229, a diagnosis of euglycemic DKA was made. He was treated according to DKA protocol and discharged. The relationship between SGLT2 inhibitors and euglycemic DKA remains to be explored; given the absence of clinically significant hyperglycemia during the presentation, a delay in diagnosis may be observed. After an extensive literature review, we introduce our case presentation in the setting of gastroparesis in comparison to previous reports and suggest future improvements in terms of early clinical suspicion for euglycemic DKA.

## 1. Introduction

**Figure 1:**
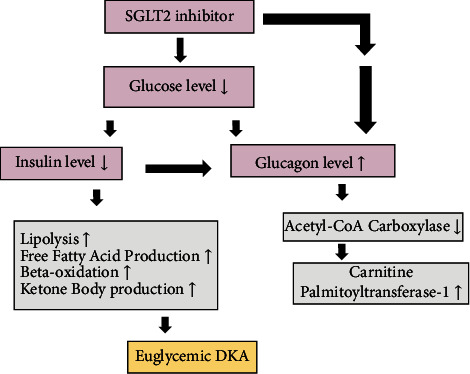
Proposed mechanism of euglycemic DKA induced by SGLT2-inhibitors.

Euglycemic diabetic ketoacidosis (euDKA) describes a syndrome that occurs in both type 1 and type 2 diabetes mellitus, characterized by euglycemia with blood glucose less than 250 mg/dL in the presence of metabolic acidosis. Metabolic acidosis is defined as an arterial pH less than 7.3, serum bicarbonate less than 18 mEq/L, and ketonemia [1]. Diabetic ketoacidosis is a life-threatening condition and must be immediately treated. Insulin deficiency and the presence of excess counterregulatory hormones such as glucagon, catecholamines, or corticosteroids result in higher ketone levels [2]. Ketone bodies contribute to metabolic acidosis and glucosuria induced by SGLT-2 inhibitors, leading to osmotic diuresis, dehydration, and hypovolemia (Figure 1) [2].

The incidence of euDKA has increased with the introduction of sodium-glucose cotransporter type 2 inhibitors (SGLT2i); its diagnosis poses a diagnostic challenge and at times delayed diagnosis due to the range of etiologies and normal blood glucose levels. The mechanism is due to a general state of starvation resulting in ketosis while maintaining normoglycemia. Triggers for euDKA can include reduced insulin doses, alcohol intake, persistent vomiting, gastroparesis, intercurrent illness, sepsis, shock, and lower food/fluid intake [1, 3].

SGLT2i's were introduced in 2013 and changed the landscape of type 2 diabetes mellitus treatment [3]. SGLT2i acts by increasing urinary glucose/sodium excretion and blocking the resorption of glucose from the proximal convoluted tubule, leading to the loss of urinary glucose to create a state of carbohydrate starvation and volume depletion [1].

Soon after their presence in the market, the US Food and Drug Administration (FDA) released a drug safety notice that warned of an increased risk of euglycemic diabetic ketoacidosis (euDKA) associated with the class of drugs. Incidence of DKA associated with SGLT2i's ranges from 0.16 to 0.76 events per 1000 patient-years in those with type 2 diabetes mellitus [1]; it is estimated that they increase the risk in type 2 diabetic patients by seven times [1].

## 2. Case Presentation

In August 2022, a 49 year-old caucasian male presented to the emergency department with complaints of epigastric abdominal pain, angina, and intractable vomiting. His past medical history is significant for type 2 diabetes mellitus diagnosed over twenty years ago, neuropathy, obesity, (BMI > 30), gastroparesis, polysubstance abuse, history of alcohol abuse-last drink 7 months ago, and depression. The patient noted that his abdominal pain was similar to his prior episodes of gastroparesis describing it as sharp and pressure-like; however, this time the abdominal pain radiated to his left chest/shoulder with associated vomiting. He admitted to seeing his endocrinologist several months ago and agreed that he was compliant with his medications.

On presentation to the emergency department, the patient was in no acute distress with vitals revealing a normal rate, moderately hypertensive (170's systolic) with no tachypnea or signs of hypoxia. Weight was 109.9 kg and BMI > 30. A physical exam revealed a mildly obese abdomen as the patient was actively vomiting into an emesis bag on arrival, along with diffuse tenderness to the abdomen mainly located near the epigastrium region without any rebound, guarding, or rigidity.

ECG in the Emergency department showed no acute changes and serial troponins were negative. No significant electrolyte abnormalities were present. Arterial blood gas demonstrated an elevated anion gap with a mildly decreased serum CO_2_ level. Reactive vs. infectious etiologies were considered as labs demonstrated elevated leukocytosis > 16 and increased neutrophils. CTA chest was negative for aortic aneurysm or dissection. The urine drug screen was positive for cocaine and cannabinoids. Chest X-ray showed mild central vascular congestion with low lung volumes. The patient was administered crystalloid fluids, morphine for pain control, hydralazine for blood pressure control, and Zofran for antinausea. The patient was admitted to the internal medicine team, and an insulin drip was started to correct his impending diabetic ketoacidosis. Dextrose was continued and beta-hydroxybutyrate levels were ordered.

**Figure 2:**
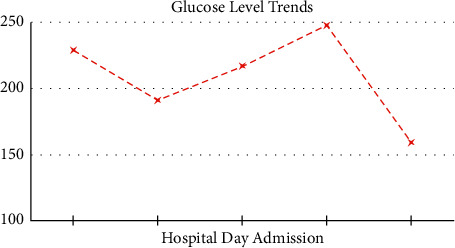
Glucose level trends during patient hospital day.

He was evaluated the following morning and reported constant epigastric abdominal pain in addition to nonbloody emesis and inability to tolerate oral fluids with resolved chest pain, fever, and chills. It was noted that he was diagnosed with gastroparesis via a gastric emptying study a few months earlier. Upon further review, blood glucose readings remained in the ranges of 150–240 (229 > 191 > 217 > 248 > 159) as seen in Figure 2. A1c was 8.1, eGFR > 60, and lactate 1.7. Arterial blood gas shows pH 7.207, PCO_2_ 27.9, and HCO_3_ 11.1 with a base excess of 16.8. The elevated beta-hydroxybutyrate level was at 3.843. The rise in chloride was likely secondary to repeated vomiting. Endocrinology evaluated the patient and advised to transition from insulin drip to subcutaneous regimen injections and also suspected patient noncompliance with medications of 100 U Lantus twice a day, Novolog 30 U AC, Jardiance 25 mg, and Ozempic 0.25 mg once a day.

Throughout the patient's hospital course, his diabetic medication regimens consisted of Lantus with a dose increase from 30 to 45 mg BID due to persistently elevated BGL (228–255), Humalog 10 units AC meals, and Levemir 100 units BID. The patient was soon able to tolerate a diet with resolved gastrointestinal symptoms, potassium stabilized at 5.0, normalized ABG with corrected AG, and was hemodynamically stabilized. Glucose at discharge was 239. The patient was then discharged on continued hospital diabetic regimen control in addition to Dicycloverine 20 mg and Zofran 4 mg. Empagliflozin was discontinued secondary to euDKA.

## 3. Discussion

Introduced in 2013, the SGLT2i's constitute a relatively new class of oral hypoglycemic agents that can be used as alternative monotherapy or adjunctive therapy for patients with diabetes mellitus who fail prior initial measures including lifestyle modifications, sulfonylureas, or metformin [4]. They are preferred in patients with existing heart failure, chronic kidney disease, or atherosclerotic vascular disease due to disease risk reduction, independently of their ability to reduce blood glucose and HbA1c levels. In addition, SGLT2i's also promote weight loss, reduce exogenous insulin requirements, and decrease blood pressure [4].

Despite the myriad of positive benefits, SGLT2i's also have been associated with increased urinary tract infections, volume depletion, and genital mycotic infections [4]. Their association with euDKA has been documented but not established; thus, this particular patient presentation helps to strengthen the possible link. The ratio of insulin to glucagon is thus altered in euDKA, resulting in ketosis [4]. Early detection of ketosis in the form of daily urine or ketone blood testing could prevent patients from quickly deteriorating [5]. Patients with moderate to large urine ketones should be advised to discontinue the SGLT2-i temporarily, contact their healthcare provider, increase hydration, and upregulate carbohydrate consumption until the ketones resolve [5]. The SGLT2-i may be restarted at a later date if appropriate.

## 4. Conclusion

In summary, we present a possible association with the use of SGLT2i's resulting in the increased risk of euDKA. Modifiable factors cannot be excluded such as compliance with medical therapy, diet, and other lifestyle modifications. In this case, the patient's concomitant history of gastroparesis may have affected the likelihood of triggering euDKA. It remains a diagnosis of exclusion yet should always be considered in any ill patient with a history of type 1 or type 2 diabetes mellitus with decreased blood glucose levels and the presence or absence of urine ketones. Currently, limited data is reported on the use of SGLT2i's ability to increase the risk of developing euDKA with more research required. Further studies will help elucidate a link between the quantity of urine ketones and SGLT2i dependence.

## Data Availability

Data sharing is not applicable.
